# Association of Internet Addiction with Family Functionality, Depression, Self-Efficacy and Self-Esteem among Early Adolescents

**DOI:** 10.3390/ijerph17238820

**Published:** 2020-11-27

**Authors:** Hsiao-Ching Chen, Jiun-Yi Wang, Ying-Lien Lin, Shang-Yu Yang

**Affiliations:** 1 Department of Healthcare Administration, College of Medical and Health Science, Asia University, Taichung 413, Taiwan; orlay626@gmail.com (H.-C.C.); jjwang@asia.edu.tw (J.-Y.W.); 2 Department of Industrial and Information Management, National Cheng Kung University, Tainan 701, Taiwan; t10025023@gm2.nutn.edu.tw

**Keywords:** internet addiction, early adolescent, gender, family functionality, money allowance, depression, self-efficacy, self-esteem

## Abstract

Early adolescents usually have worse self-control and poor time management abilities. They are a higher-risk group for Internet addiction than older adolescents or adults. This study aims to explore the prevalence of Internet addiction and associated factors in early adolescents. Participants included 451 fifth and sixth-grade students in Central Taiwan. This study adopted a cross-sectional design and a structured questionnaire that consisted of demographics, Young’s Internet Addiction Test, the Centre for Epidemiological Studies-Depression scale, the General Self-Efficacy Scale and the Rosenberg Self-Esteem Scale. The participants were 50.8% male, and the mean age was 11.35, with a range of 10.33–12.92 years. A total of 33.7% of all participants were prone to Internet addiction. The results showed that participants who were male and had high money allowance, poor family atmosphere, parents who did not limit Internet usage time, high depression, low self-efficacy and low self-esteem were more prone to Internet addiction. A multiple logistic regression analysis revealed that being male, having parents who did not limit Internet usage time and higher degrees of depression were the associated factors of Internet addiction in early adolescents. The phenomenon of Internet addiction among early adolescents seems to be increasing. Improving family functionality and individual mental health may be effective ways to reduce Internet addiction.

## 1. Introduction

The Internet provides access to the world and has changed people’s habits while becoming a major part of daily life [1]. However, problematic Internet use has emerged as a major public health problem and has received increasing empirical attention in recent years [2,3,4]. As a result of this topic’s complex nature, scholars have coined different terms to describe this phenomenon, such as problematic Internet use [5], pathological Internet use [2], compulsive Internet use [6] and Internet disorders [7]. Therefore, in this study, a maladaptive pattern of Internet use that leads to clinically significant impairment or distress is defined as Internet addiction (IA) [8,9].

According to a 2012 study in Europe, the prevalence of IA was reported at 5.4% in Italy, 8.2% in Greece and 18.3% in England [10]. A study of Southern U.S. university students found that about a quarter met the criteria for Internet dependence [11]. A prevalence of IA ranging from 2.4% to 37.9% was also reported in Asia amongst adolescents and young people [4]. These rates are generally high and sometimes alarming. Internet use has grown worldwide in all age groups, and the number of children and adolescent Internet users has increased dramatically [10,12,13,14]. Reportedly, Internet use at an early age could lead to escalated addiction risks [15]. Cognitively, early adolescents have the capacity to expand their intellectual interests, but they usually have lower levels of self-control and poor cognition [16]. They often fall into higher-risk groups for IA than older adolescents or adults [17]. Therefore, identifying the causes of IA in early adolescents is important for the development of preventive strategies.

The cognitive behavioural model has received empirical support to explain the aetiology, development and consequences of IA [2,18,19,20]. More recent formulations of the cognitive behavioural model have proposed a series of related components, including a preference for online social relationships, mood regulation through the Internet, deficient self-regulation and negative consequences [2,19,20,21,22]. Furthermore, most of these components reflect the various physiological, psychological and environmental problems that occur during adolescence [21].

Scholars have characterised IA by the appearance of various negative consequences in different individuals. Many studies have been conducted to identify the factors associated with IA through documented demographic variables. Male adolescents were more prone to IA in several studies [23,24,25]. However, the existing scientific literature does not provide a consistent conclusion. For example, Yang et al. [26] found that female adolescents as compared to males exhibited significantly higher degrees of smartphone dependence among junior college adolescents. There is no conclusive agreement on gender in IA, which suggests the need to further investigate this issue [19,20]. 

In addition, recent studies have indicated that excessive Internet use is related to family functionality [27,28]. It is emphasised that family ambience is a determining factor of an adolescent’s overall Internet use [29]. Especially at a younger, pubescent age, relationships in the family widely affect children’s physiological and mental growth. In situations where adolescents feel a lack of family functionality, they attempt to fill this absence with virtual love and relationships [30]. Young people may establish relationships via the Internet where they can find temporary intimacy, a sense of belonging and involvement [28].

IA has been confirmed to be associated with various psychological problems [31,32,33]. Depression is a positive predictor of adolescent Internet addiction [25], and there is strong evidence that individuals with higher levels of depression are more likely to develop IA [30,31,34,35]. A preference for online social interactions stems from the belief that relationships online are safer, more comfortable and less threatening than face-to-face interactions [18,36]. However, when Internet use provides relief for problems such as depression, it is likely to become addictive [37,38,39].

In response to this phenomenon, some scholars have proposed self-efficacy as a significant predictor of Internet addiction [22,40,41]. Self-efficacy addresses an individual’s perception of control over life events and is predictive of a person’s goals and performance [42]. Deficient self-efficacy includes both immoderate thoughts and an individual’s inability to regulate Internet connection behaviours [43,44]. In addition, people with low self-efficacy tend to gain a sense of accomplishment by investing in the Internet. At the same time, they provide cognitive justification of their compulsive behaviours related to the Internet and their obsession with connecting via the Internet [45].

Self-esteem is another psychological trait that has been associated with IA in several studies. Self-esteem is described as the evaluation that people have of themselves and how they feel about themselves in all situations [46,47]. Individuals who lack self-confidence in general have low self-esteem. Thus, they are less motivated to communicate in the real world, because they expect to fail. Such individuals rather prefer online social interactions, because they think they can interact better and more confidently there [39,48,49,50]. As a result, adolescents who have low self-esteem could become Internet-addicted users.

Therefore, the purpose of this study is to explore the associated factors of IA in early adolescents. Based on the findings of our literature review, we arrived at the following hypotheses:

**Hypothesis** **1.** *There was a gender difference in IA.*

**Hypothesis** **2.** *Good family functionality was negatively associated with IA.*

**Hypothesis** **3.** *Higher levels of depression were positively associated with IA.*

**Hypothesis** **4.** *Lower self-efficacy was positively associated with IA in early adolescents.*

**Hypothesis** **5.** *Lower levels of self-esteem were positively associated with IA.*

## 2. Materials and Methods 

This study used a structured questionnaire to justify hypotheses through a cross-sectional design [51,52].

### 2.1. Participants

Before recruiting the study participants, the sample size was calculated by using G*Power V3.1. An alpha error probability (α err prob) of 0.05, a power (1-β err prob) of 0.8 and a medium effect size f^2^ of 0.15 were also considered [53]. A power analysis indicated the need for at least 240 participants. Early adolescence is broadly believed to be between 10 and 13 years of age [16,54]. In order to examine IA in the initial stage of puberty, we chose 5th and 6th-grade students (the mean age was 11.35) with an age range of 10.33–12.92 years from an elementary school in Central Taiwan. Changes during puberty make 5th and 6th-grade students vulnerable to a variety of mental health problems [55], so it is necessary to pay particular attention to the development of this group.

### 2.2. Instrument

#### 2.2.1. Demographics and Family Functionality

Demographics (gender, age and body mass index) and family functionality (money allowance per week (New Taiwan Dollar); parental limit of Internet usage time; family atmosphere and living with parents, grandparents or relatives) were recorded on the questionnaire. At the same time, Internet usage was surveyed in this study, including daily Internet usage hours on the weekday/weekend in the previous week and a multiple-choice question about the most common uses of the Internet for academic needs, web surfing, audio-visual web entertainment, the usage of social media, online shopping and video games.

#### 2.2.2. IA

IA was measured using the 20-item Chinese version of Young’s Internet Addiction Test (IAT) [9]. The Chinese version of the IAT possesses favourable reliability and validity [56,57]. Items were rated on 5-point scales, ranging from 1 (rarely) to 5 (always), and a final score was calculated, with higher scores indicating more Internet addictive behaviours. According to Young’s classification standard [8], normal range: 0–30, mild addiction: 31–49, moderate addiction: 50–79 and severe addiction: 80–100. Kishore [58] proposed the cut-off criteria: all those who scored >30 were considered to have IA. This study categorised those with IAT scores ≥31 as the Yes-IA group and those who scored ≤30 as the No-IA group. Furthermore, the internal consistency of Cronbach’s alpha for the IA items was good (α = 0.85).

#### 2.2.3. Depression

The level of depression was assessed using the Chinese version of the 20-item Centre for Epidemiological Studies-Depression scale (CES-D), which is a commonly used, self-reported instrument [59]. The Chinese version of the CES-D presents advantageous reliability and validity [60,61]. Participants rated how often they experienced the symptoms in the previous week on a 4-point Likert scale, ranging from 1 (less than 1 day) to 4 (5–7 days). The scoring of positive items was reversed, and the total sum ranged from 20 to 80, with higher scores indicating more symptoms of depression. The depression measure in this study had good reliability (Cronbach’s alpha = 0.86).

#### 2.2.4. Self-Efficacy

The Chinese version of the General Self-Efficacy Scale (GSES) was used in this study to assess participants’ abilities to perform in unexpected situations or surprising events across 10 items rated on a 4-point scale ranging from 1 (not at all true) to 4 (exactly true). The Chinese version of the GSES shows excellent reliability and validity [62,63]. A sum of all scores yields ranged from 10 to 40. Higher scores indicated a higher level of self-efficacy [64]. The present study about self-efficacy showed a good reliability coefficient of 0.89.

#### 2.2.5. Self-Esteem

The Chinese version of the Rosenberg Self-Esteem Scale (RSES) was used to provide an evaluation of a participant’s self-esteem [65] using a 10-item scale. The Chinese version of the RSES dominates good reliability and validity [63,66]. All statements were rated on a 4-point Likert scale ranging from 1 (strongly agree) to 4 (strongly disagree). The scale measures both positive and negative feelings about the self. Low self-esteem responses are “disagree” or “strongly disagree” on positive items and “strongly agree” or “agree” on negative items. The scale can also be summed by totalling the individual 4-point items after reverse-scoring the negatively worded items. The scale ranges from 10 to 40. Lower scores indicate higher levels of self-esteem. The internal consistency of this scale was good in the present study (α = 0.81).

### 2.3. Procedure

Permission for the in-school survey was obtained from the school principals. Prior to the administration of the measures, all participants were told about the purposes of the study. All participation was voluntary, and letters of consent were obtained from the participants and their guardians. All respondents were assured of anonymity and confidentiality. Data were collected in June 2020. All structured questionnaires were distributed to the participants in classroom settings and were collected onsite after 30 min. The questionnaires were self-reported. It was ensured that no person other than the researcher and the study participants were present in the classroom during the 30-min period to avoid any bias, influence or hesitancy. The inclusion criteria included having the ability to communicate in Mandarin and completing the consent forms. The exclusion criteria in this study included having a reading disorder or being in a special education class. Among the 470 students invited to participate in the study, 5 gave up the test on the spot, and 14 test sheets were not completed. The investigations were carried out following the rules of the Declaration of Helsinki of 1975, revised in 2013 [67]. The ethical approval for the study and the consent procedure were approved by the Jen Ai Hospital Human Research Ethics Committee (JAH HREC-109-27).

### 2.4. Statistical Analysis

First, descriptive statistics (e.g., means/M, standard deviation/SD and percentages) were presented about demographics, family functionality, Internet usage, depression, self-efficacy and self-esteem. After this, the following tests were used for the bivariable analysis. Associations between demographics, family functionality, depression, self-efficacy, self-esteem and IA, with individual students as No-IA or Yes-IA, were tested using a chi-square test for categorical data and a Mann-Whitney U test for numerical data. Finally, multiple logistic regression was performed to determine the effects of independent variables that were significantly associated with IA to predict the dependent variable of the IAT scores. Their odds ratios (OR) and their respective 95% confidence intervals (CIs) were derived. All statistical analyses were performed using SPSS v25.0 (IBM Corp., Armonk, NY, USA), and statistical significance was set at a level of *p* < 0.05.

## 3. Results

### 3.1. Descriptive Statistics of Demographics, Family Functionality, Internet Usage, Depression, Self-Efficacy, Self-Esteem and IAT

Of the 470 students invited to participate in the study, five gave up the test on the spot, 14 were unfinished and 451 completed the questionnaires, giving a response rate of 96%. Table 1 shows the distribution of participants in terms of demographics, family functionality, depression, self-efficacy and self-esteem. The sample was comprised of more males (50.8%), the mean age was 11.35 (SD = 0.56), with a range from 10.33 to 12.92 years, the average body mass index (BMI) value was 19.10 kg/m^2^ (SD = 4.28) and the mean money allowance per week was 80.97 (SD = 135.5) NTD (New Taiwan Dollar). More than 80% of parents limited Internet usage time (81.2%). Around three-quarters of participants’ family atmospheres were harmonious (25.1%) or very harmonious (48.1%). Nearly 70% participants lived with their parents (68.5%). In the survey of participants’ daily Internet usage, the time spent on the Internet was 1.69 (SD = 1.11) hours during the week and 2.74 (SD = 1.49) hours during the weekend. The most common uses of the Internet were audio-visual web entertainment (88.9%), usage of social media (51.7%), academic needs (39%), web surfing (33.7%), video games (22.8%) and online shopping (9.1%). The IAT of the participants had an average score of 29.21 (SD = 8.41). More than one-third (33.7%) of the participants belonged to Yes-IA, who had the average score of 38.68 (SD = 7.55). The participants had a mean score about the CES-D of 29.33 (SD = 7.65), GSES of 25.36 (SD = 6.30) and RSES of 19.5 (SD = 4.91).

### 3.2. Associations of Demographics, Family Functionality, Depression, Self-Efficacy and Self-Esteem with IAT

The results of the chi-square analysis and the Mann-Whitney U test showed that there were significant differences between the No-IA or Yes-IA groups when considering the factors of demographics, family functionality, depression, self-efficacy and self-esteem. More statistical information can be found in Table 2. In the Yes-IA group, the proportion of males was significantly higher than that of females (38.4% vs. 28.8%, *p* = 0.031). The money allowance per week that participants in the Yes-IA group had was significantly higher than those in the No-IA (106.28 NTD vs. 68.10 NTD, *p* = 0.018). The family atmosphere of the Yes-IA participants was significantly less harmonious than that of the No-IA participants (3.92 vs. 4.29, *p* < 0.001). Among the Yes-IA participants, the proportion of parents who did not limit Internet usage time was significantly higher than the proportion of parents who did limit it (56.5% vs. 28.4%, *p* < 0.001). The CES-D scores in the Yes-IA participants were higher than those in No-IA participants (32.39 ± 8.87 vs. 27.78 ± 6.43, *p* < 0.001), which means the depression of the Yes-IA group was higher than that of the No-IA group. The scores of the GSES in the Yes-IA participants were lower than those in the No-IA participants (24.24 ± 6.05 vs. 25.93 ± 6.36, *p* = 0.007), meaning that the self-efficacy of the Yes-IA group had poorer status than that of the No-IA group. The RSES scores in the Yes-IA participants were higher than those for the No-IA participants (20.89 ± 4.94 vs. 18.79 ± 4.75, *p* < 0.001), which means the self-esteem of the Yes-IA group was lower stage than that of the No-IA group.

### 3.3. Exploring the Associated Factors of IA by Multiple Logistic Regression

Multiple logistic regression was performed to determine the effects of the variables of gender, money allowance per week, family atmosphere, parental limit of Internet usage time, CES-D, GSES and RSES on the likelihood of having IA, the results of which are shown in Table 3. The result of the multiple logistic regression analysis revealed that being male (female vs. male, OR = 0.60, 95% CI = 0.39~0.92, *p* = 0.02), having more severe depression (OR = 1.07, 95% CI = 1.03~1.11, *p* < 0.001) and having parents who did not limit Internet usage time (Yes vs. No, OR = 0.31, 95% CI = 0.18~0.52, *p* < 0.001) were positively associated with IA, accounting for 20.7% of the variance in the model (*p* < 0.001) about the participants.

## 4. Discussion

### 4.1. Prevalence of IA and Internet Usage

The results of this study found that the average IAT score of the participants was 29.21, indicating that they had almost reached the baseline of IA. According to the result of the study, the participants mostly had limited time on the Internet by their parents. Consistent with other studies [68,69], parental restrictive mediation of Internet use for children was needed to prevent IA. 

Unexpectedly, this study found that one in three adolescents were addicted to the Internet. They are mildly addicted to the Internet according to Young’s classification standard. The prevalence of excessive Internet use is significant among early adolescents in Taiwan, which conforms with the global trend [3,70]. Recent reports have determined that the percentage of Internet use skyrocketed from childhood to early adolescence [3]. The Internet’s use for a variety of purposes, its easy accessibility and its implied or explicit encouragement may result in more young Internet addicts [71]. Moreover, in the present study, the most common uses of the Internet were for audio-visual web entertainment and social media. These factors caused positive emotion and induced reinforcement to increase Internet time [70,72,73]. Moreover, the participants’ daily Internet usage time (i.e., 1.69 h during weekdays and 2.74 h on weekends) in this study was similar to that in previous studies, in which 39.4% of adolescents reported spending at least 2 h online on a normal school day [17]. In accordance with the developmental mechanism of IA, longer Internet use times were associated with IA risk [74,75]. We identified IA as a significant public health threat, and countries around the world need to prevent IA from becoming worse by supporting education, research and treatment [76].

### 4.2. Associations Between Gender and IA

Regarding the multiple logistic regression analysis, gender had a significant correlation with IA. Males in early adolescence are more likely to become addicted to the Internet, which supports Hypothesis 1 and is consistent with some IA studies [23,24]. Cho, Sung, Shin, Lim and Shin mentioned that male students use the Internet as a channel for leisure and entertainment, which are accompanied by pleasant emotions, leading to a cycle of IA where the longer the use, the happier the feeling [77]. Furthermore, Shek and Yu also determined that boys tend to be enthusiastic about Internet technology development or learning about Internet applications [27]. Therefore, teachers and parents need to pay attention to the gender differences of IA and provide appropriate counselling and assistance to understand male needs during the adolescent period [78].

### 4.3. Associations Between Family Functionality and IA

This study found that the participants with a higher money allowance had a greater chance for IA. Premsingh and PV mentioned that Internet behaviour is a form of consumerism, including software, hardware, Internet fees, etc., which require considerable financial support to operate and use [79]. Research by Yao et al. mentioned that, when a student can use more money on their own, they are more able to conduct Internet behaviour through money transactions without parental consent [30]. Therefore, parents need to understand the financial needs of early adolescents, give them an appropriate money allowance amount and assist them in proper money use in order to reduce IA.

This study also found that the more harmonious the family atmosphere, the less likelihood for IA. Simultaneously, Shek and Yu also found that a good family atmosphere had a significant impact on avoiding dangerous behaviours and IA of adolescents [27]. On the contrary, a bad family atmosphere will lead children to get involved in the virtual world in order to obtain temporary emotional support and a sense of belonging [30]. Consequently, parents who establish a good family atmosphere can decrease the IA of their early adolescents.

Furthermore, as a result of the multiple logistic regression analysis, parental intervention on the Internet usage time will reduce students’ IA. The home is where early adolescents use the Internet most often. At the same time, this study also showed nearly 70% of participants lived with their parents. Parental monitoring may deter adolescents from becoming addicted to Internet use [80]. Gündüz and Şahin also mentioned that parents should give verbal warnings and even ban Internet access to prevent excessive Internet use among children aged 10–18 [81]. Thus, good family functionality would decrease early adolescents prone to IA, which supports Hypothesis 2. The better approaches focus on the parent-child involvement and high-quality communication for preventing early adolescents from becoming addicted to the Internet [29].

### 4.4. Association of IA with Depression, Self-Efficacy and Self-Esteem

The multiple regression analysis revealed that depressive symptoms were the most predictable pathology for Internet addiction, which supports Hypothesis 3. As pointed out earlier, there is empirical data supporting the idea that depressive people find comfort and consolation in cyberspace. Cho et al. conducted IA research on childhood psychopathology and contended that depression is related to inappropriate Internet use [77]. Lee et al. asserted that students may use the Internet to distract themselves from their feelings of depression, slow their emotions and avoid facing unhappy situations [34]. These findings suggest that Internet use is reinforced by pleasurable thoughts and feelings that occur while using the Internet [82]. Thus, students feel depressed and use the Internet to escape negative feelings [25].

This study also showed that the lower the self-efficacy, the higher the chance of IA in early adolescents, which supports Hypothesis 4. Consistent with these results, self-efficacy was found to be negatively related to IA [37,40,45]. People with higher self-efficacy strongly believe in their ability to influence their lives [83]. Adolescents with high self-efficacy may have better control over their own time and are better able to properly plan their daily lives [44]. On the contrary, adolescents with low self-efficacy are weak in scheduling skills and are more likely to indulge in online games and social media that can help them escape the stress of real life [84]. Thus, interventions focused on increasing self-efficacy can be useful in decreasing symptoms of Internet addiction [44].

In addition, this study pointed out that students with lower self-esteem are more likely to become addicted to the Internet, which supports Hypothesis 5. This result is the same as that of Fioravanti et al., who conducted an analysis of IA in adolescents and found that self-esteem was related to IA [48], as well as Kim and Davis, who proposed that low self-esteem can predict problematic Internet use [35]. People with higher self-esteem believe that they can deal with difficulties and do not need to seek solace in the virtual world [85]. On the contrary, the use of Internet is associated with perception as a way of coping with the compensation of some deficiencies in low self-esteem adolescents [86]. Accordingly, giving students self-esteem training and courses may promote normal Internet use behaviour.

In summary, early adolescents with depression, low self-efficacy or low self-esteem may struggle in the real world. Three highly significant reasons exist for resorting to the Internet as a form of escape [21,38]. First, some early adolescents can freely create their own identities online, with the features they want to possess, both physical and psychological [40]. Second, anonymity constitutes another captivating characteristic of the Internet, because early adolescents can freely express their opinions or attitudes without the fear of rejection [43]. Third, young people also find interaction and support online, which matter to adolescents who often do not receive adequate social support in their surroundings [31]. Future prevention programmes should focus on psychosocial competencies and specific skills that help early adolescents to develop critical thinking on their own relationship with the Internet and a stronger commitment to understanding the meanings of their own involvement [87].

### 4.5. Limitations

The results of this study should be considered in light of its limitations. First, participants were all from the same school, and this study was a cross-sectional research, which makes us more conservative when making research inferences. Longitudinal studies will be needed to adequately examine the direction of the effects. Second, the sample was restricted to early adolescents in Taiwan, limiting the applicability of our findings in Western countries. Third, the use of convenience sampling limited the generalisability and induced bias. Fourth, the influential factors were perceived solely based on adolescents’ self-reported data, and there was a lack of information from the parents’ and teachers’ perspectives. Fifth, the pressure to provide socially desirable responses concerning IA may make participants unwilling to reveal their true selves, even in anonymous questionnaires. Sixth, the study could not include all possible factors. Further studies should attempt to determine additional factors.

## 5. Conclusions

This study contributes to the current literature on IA by exploring the associated factors of IA that reveal the relationships between depression tendencies, low self-efficacy, low self-esteem and IA in early adolescents. In addition, this study found that participants’ money allowance per week, parental limitation of Internet usage time and family atmosphere were also influencing factors in IA. In summary, psychological variables are the motivator, and family functionality is an inhibitor in adolescents’ IA. Early adolescence is the initial stage at which young people experience significant changes biologically, psychologically and socially. Parents should play a protective role in early adolescents’ behaviours, conduct appropriate online time and improve the parent–child communication proficiency to achieve healthy family functionality. These findings can benefit psychologists, school counsellors, teachers and parents in the development of prevention and intervention programmes for IA.

## Figures and Tables

**Table 1:** Descriptive statistics of demographics, family functionality, depression, self-efficacy, self-esteem and IAT (*n* = 451).

	N	%	Mean	SD
Gender				
Male	229	50.8		
Female	222	49.2		
Age			11.35	0.56
BMI (kg/M^2^)			19.10	4.28
Money allowance per week (NTD)			80.97	135.50
Family atmosphere			4.17	0.94
Very discordant			6	1.3
Discordant			8	1.8
Ordinary			107	23.7
Harmonious			113	25.1
Very harmonious			217	48.1
Living with				
Parents	309	68.5		
Grandparents	134	29.7		
Relatives and friends	8	1.8		
Did parents limit Internet usage time				
No	85	18.8		
Yes	366	81.2		
IAT score			29.21	8.41
No-IA Group & score	299	66.3	24.39	2.99
YES-IA Group & score	152	33.7	38.68	7.55
CES-D			29.33	7.65
GSES			25.36	6.30
RSES			19.50	4.91

BMI: Body mass index, IAT: Internet Addiction Test, CES-D: Centre for Epidemiological Studies -Depression, GSES: General Self-Efficacy Scale and RSES: Rosenberg Self-Esteem Scale.

**Table 2:** Associations of demographics, family functionality, depression, self-efficacy and self-esteem with IAT (*n* = 451).

	Internet Addiction				
	No (*n* = 299)		Yes (*n* = 152)		*p*-value ^b^
	N	%	N	%	
Gender					0.031 *
Male	141	61.6%	88	38.4%	
Female	158	71.2%	64	28.8%	
Age ^a^	11.31	0.57	11.41	0.53	0.095
BMI (kg/M^2^) ^a^	18.88	4.20	19.51	4.40	0.126
Money allowance per week (NTD) ^a^	68.10	114.15	106.28	167.41	0.018 *
Family atmosphere ^a^	4.29	0.92	3.92	0.92	<0.001 *
Living with					0.107
Parents	204	66.0%	105	34.0%	
Grandparents	90	67.2%	44	32.8%	
Relatives and friends	5	62.5%	3	37.5%	
Did parents limit Internet usage time					<0.001 *
No	37	43.5%	48	56.5%	
Yes	262	71.6%	104	28.4%	
CES-D ^a^	27.78	6.43	32.39	8.87	<0.001 *
GSES ^a^	25.93	6.36	24.24	6.05	0.007 *
RSES ^a^	18.79	4.75	20.89	4.94	<0.001 *

BMI: Body mass index, IAT: Internet Addiction Test, CES-D: Centre for Epidemiological Studies -Depression, GSES: General Self-Efficacy Scale and RSES: Rosenberg Self-Esteem Scale. ^a^ Data were presented as mean ± SD. ^b^ Chi-square test was used for categorical data. Mann–Whitney U test was used for numerical data. * *p* < 0.05.

**Table 3:** Exploring the associated factors of Internet addiction by multiple logistic regression (*n* = 451).

	OR	95% CI		*p*
		Lower	Upper	
Gender (ref. Male)				
Female	0.60	0.39	0.92	0.020 *
Money allowance per week (10 NTD)	1.01	1.00	1.03	0.096
Family atmosphere	0.81	0.64	1.02	0.072
Did parents limit Internet usage time? (ref. No)				
Yes	0.31	0.18	0.52	<0.001 *
CES-D	1.07	1.03	1.11	<0.001 *
GSES	0.99	0.96	1.03	0.762
RSES	1.02	0.96	1.08	0.627

CES-D: Centre for Epidemiological Studies-Depression, GSES: General Self-Efficacy Scale and RSES: Rosenberg Self-Esteem Scale; * *p* < 0.05. OR: odds ratio.
